# Infection image: reoccurrence of Kaposi`s sarcoma after SARS-CoV-2 mRNA vaccination in an HIV-infected patient

**DOI:** 10.1007/s15010-023-02121-9

**Published:** 2023-12-06

**Authors:** Florian Hitzenbichler, Markus Weber, Bernd Salzberger

**Affiliations:** 1 Department of Infection Prevention and Infectious Diseases, University Hospital Regensburg, Franz-Josef-Strauß-Allee 11, 93053 Regensburg, Germany; 2 Department of Sarcoma Surgery, Barmherzige Brueder Regensburg, Prüfeninger Str. 86, 93049 Regensburg, Germany; 3 Department of Infection Prevention and Infectious Diseases, University Hospital Regensburg, Franz-Josef-Strauß-Allee 11, 93053 Regensburg, Germany

A 48-year-old male Caucasian patient was diagnosed with HIV infection and Kaposi`s sarcoma (KS) on his right foot sole and lower leg in 2008 (CDC classification C2). Antiretroviral combination therapy was started and both KS lesions showed spontaneous remission within six months.

In May 2021 he received the first dose of the SARS-CoV-2 vaccine mRNA-1273 without any adverse effects. HIV viral load was undetectable and CD4 count was 1080/µl (40%) when he presented for his scheduled appointment in May 2021.

In June 2021 a second dose of mRNA-1273 was given and approximately one week later he noticed two small flat, dark lesions on the sole of his right foot.

**Fig. 1:**
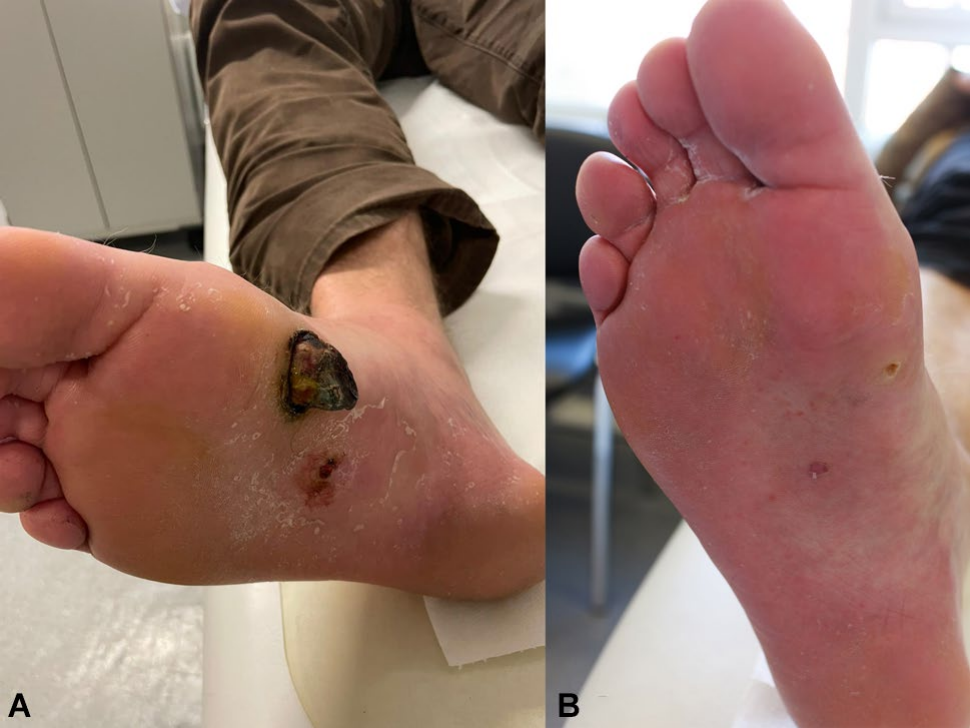
**A** Kaposi’s sarcoma lesion before resection. **B** Follow up 2 monts later

In December 2021 he received a SARS-CoV-2 booster dose (BNT162b2). Approximately one week later one of the lesions on his sole progressed to an ulcerative tumor (Fig. 1A).

He presented at the local sarcoma center where the tumor was resected. Histology confirmed KS.

On follow-up in February 2022 HIV viral load was not detectable and CD4 count was 791/µl. Staging with a CT scan of the thorax and abdomen and upper and lower GI-endoscopy did not show any signs of other KS lesions.

After resection of KS, the lesions did not reappear (Fig. 1B). Human Herpesvirus 8 (HHV-8) DNA was detectable in blood samples from February 2022 but was not detectable in November 2020 (before SARS-CoV-2 vaccination).

After both BNT162b2 and mRNA-1273 vaccinations cases of herpes zoster were noted [1, 2]. Clinical apparent reactivations of other herpes viruses have also been reported [3, 4].

Our patient´s case with HHV-8 reactivation and the development/reoccurrence of KS lesions in association with a three-dose series of SARS-CoV-2 mRNA vaccination suggests HHV-8 reactivation and KS reoccurrence as an adverse event of mRNA vaccination. Surgical resection in our case was successful for local control, but should not be regarded as the treatment of choice for other regions.
